# Omics-based biomarkers of immune-related organ toxicities associated with immune checkpoint inhibitors: a scoping review and evidence map

**DOI:** 10.3389/fimmu.2026.1945286

**Published:** 2026-09-22

**Authors:** Jianfei Lu, Yanli Yang

**Affiliations:** 1 Faculty of Medicine, University of Macau, Macao, Macao SAR, China; 2 Tianjin Key Laboratory of Clinical Multi-Omics, Tianjin, China; 3 Department of Intensive Care Unit, National Cancer Center/National Clinical Research Center for Cancer/Cancer Hospital, Chinese Academy of Medical Sciences & Peking Union Medical College, Beijing, China

**Keywords:** evidence map, high-throughput omics, immune checkpoint inhibitors, immune-related adverse events, microbiome, scoping review, single-cell omics, transcriptomics

## Abstract

**Background:**

Immune checkpoint inhibitors (ICIs) can cause immune-related adverse events (irAEs) across multiple organs. High-throughput omics approaches may help characterize susceptibility, molecular mechanisms, diagnostic features, and monitoring markers related to irAEs; however, evidence remains dispersed across platforms, clinical applications, and toxicity phenotypes.

**Objective:**

To map original human evidence in which high-throughput omics approaches were directly linked to irAE susceptibility, occurrence, severity, diagnosis, longitudinal monitoring, clinical course, recovery, response to irAE-directed treatment, or mechanistic characterization. Methods: This scoping review and evidence map followed a registered protocol and was informed by PRISMA-ScR and JBI guidance. PubMed/MEDLINE, Scopus, Web of Science Core Collection, and Embase were searched from January 1, 2014, with the final database search completed on July 9, 2026. Eligible studies involved ICI-exposed patients or human biospecimens, implemented high-throughput genomics/statistical genetics, transcriptomics, proteomics, metabolomics/lipidomics, microbiome/metagenomics, single-cell or spatial omics, immune-repertoire sequencing, or integrated multi-omics approaches, and directly evaluated an irAE outcome. Genome-wide statistical-genetic studies were retained as a conditional evidence category. Targeted single-marker studies, routine laboratory biomarkers, efficacy-only omics analyses, non-ICI populations, non-original reports, case reports, and preclinical-only omics studies were excluded.

**Results:**

The searches identified 4,669 records. After removal of 1,151 duplicates, 3,518 unique records were screened and 433 reports were sought for retrieval. Thirty-two reports could not be retrieved for full-text assessment. Of 401 reports assessed in full text, 315 were excluded and 86 studies were included. Omics domains were non-mutually exclusive: transcriptomics was used in 50 studies, single-cell/spatial omics in 31, proteomics in 20, microbiome/metagenomics in 20, immune-repertoire sequencing in 16, genomics/statistical genetics in 14, and metabolomics/lipidomics in 6. Forty-seven studies contributed to two or more omics domains in the platform audit; after accounting for overlapping analytical modalities, 35 studies met the predefined criteria for true multi-omics integration involving independent molecular layers. Forty-five studies addressed mixed or general irAEs; among organ-specific studies, myocarditis/cardiovascular toxicity (n=10), pneumonitis/lung toxicity (n=9), and gastrointestinal/colitis toxicity (n=8) were most frequent.

**Conclusions:**

The high-throughput omics literature directly evaluating irAEs is substantially smaller than the broader biomarker literature and is dominated by transcriptomic and single-cell approaches. Most evidence remains exploratory, with limited independent assessment of predefined models or signatures, incomplete coverage of endocrine, renal, neurologic, hematologic, pancreatic, and musculoskeletal toxicities, and substantial gaps between molecular discovery and clinical implementation. Prospective multicenter cohorts, standardized irAE phenotyping, longitudinal sampling, and independent validation are required to support clinical implementation. Future studies integrating multiple molecular layers with advanced computational approaches may improve biomarker discovery and individualized risk stratification but require transparent development and rigorous validation.

**Systematic review registration:**

https://osf.io/g79cv, identifier g79cv.

## 1 Introduction

Immune checkpoint inhibitors (ICIs) targeting programmed cell death protein 1 (PD-1), programmed death ligand 1 (PD-L1), cytotoxic T-lymphocyte-associated protein 4 (CTLA-4), and other inhibitory immune pathways have become integral to the treatment of a broad range of malignancies. By restoring antitumor T-cell activity, these agents can produce durable disease control, but the same immune activation can disrupt peripheral tolerance and cause immune-related adverse events (irAEs). irAEs may affect almost any organ system, vary from mild self-limited inflammation to rapidly fatal tissue injury, and may occur during treatment or after ICI discontinuation.

The clinical heterogeneity of irAEs complicates prediction, diagnosis, and monitoring. Common manifestations include dermatitis, colitis, pneumonitis, hepatitis, and thyroid dysfunction, whereas myocarditis, neurologic syndromes, nephritis, myositis, hematologic toxicities, and pancreatic injury are less frequent but may be life-threatening. Current management relies on symptoms, routine laboratory tests, imaging, histopathology, and exclusion of competing diagnoses. These approaches are essential but are often reactive and may not identify susceptible patients before clinically significant injury. Conventional clinical factors, including age, sex, tumor type, pre-existing autoimmunity, and treatment regimen, do not fully explain inter-individual risk (1–5).

High-throughput omics technologies allow these questions to be examined across several molecular layers. Genome-wide and exome-wide analyses can identify inherited or acquired susceptibility; transcriptomic, single-cell, and spatial methods can resolve tissue- and cell-state programs; proteomic, metabolomic, and lipidomic platforms can characterize circulating or tissue-level molecular responses; microbiome and metagenomic analyses can assess host-microbial interactions; and immune-repertoire sequencing can quantify clonal T-cell and B-cell dynamics. Integrated analyses may connect these molecular layers to irAE onset, severity, organ involvement, treatment response, and recovery. Importantly, omics studies may contribute to different stages of biomarker development: some aim to identify susceptibility, diagnostic, monitoring, or prognostic features, whereas others primarily provide mechanistic insights that may support future biomarker development but do not yet represent clinically validated biomarkers. For this review, that breadth distinguished omics studies from routine laboratory measurements, single cytokines or autoantibodies, limited targeted genotyping, small conventional flow-cytometry panels, and other single-marker assays.

Previous reviews have catalogued broad clinical, serologic, immune, genetic, and microbial candidate biomarkers for irAEs (1–5), while other syntheses have focused specifically on the gut microbiome and ICI toxicity (6, 7). However, broad biomarker reviews often combine high-throughput omics with conventional or targeted assays, making it difficult to determine the size, distribution, and maturity of high-throughput omics evidence directly linked to irAEs. The literature is also fragmented by organ phenotype, cancer type, biospecimen, analytical platform, sampling time point, outcome definition, and validation strategy. Unlike previous reviews that combine conventional biomarkers with omics-derived candidates or focus on individual toxicity domains, the present review specifically maps high-throughput human omics evidence directly linked to irAE outcomes and evaluates its distribution and translational maturity at the study level.

Because the field spans heterogeneous omics platforms, biospecimens, study designs, analytical workflows, phenotypes, and validation strategies, a scoping review and evidence-map approach was selected to characterize the breadth, distribution, and maturity of the evidence rather than to estimate pooled effect sizes. We asked (1): which omics platforms and organ toxicities have been studied (2); how omics data have been used for risk prediction, diagnosis, longitudinal monitoring, mechanistic characterization, response to irAE-directed treatment, or recovery assessment (3); which biospecimens and study designs predominate; and (4) where evidence remains limited by small cohorts, cross-sectional sampling, heterogeneous phenotyping, or lack of independent validation. This eligibility rule separated systems-level molecular evidence from the much larger literature on conventional candidate biomarkers.

## 2 Methods

### 2.1 Study design and registration

We conducted a scoping review and prepared an accompanying evidence map. The methodology followed the Joanna Briggs Institute approach to scoping reviews, and reporting was guided by the PRISMA Extension for Scoping Reviews (PRISMA-ScR) (8) and JBI methodological guidance (9, 10). The protocol was prospectively registered on the Open Science Framework on May 29, 2026: Lu J, Yang Y. Omics-Based Biomarkers of Immune-Related Organ Toxicities Associated with Cancer Immunotherapy: A Scoping Review and Evidence Map (https://osf.io/g79cv). The OSF record was subsequently updated to document methodological clarifications and the completed four-database search, whose final database search was performed on July 9, 2026. The main clarifications concerned the operational definition of eligible high-throughput omics research, explicit handling of overlapping omics modalities, and conditional inclusion and interpretation of genome-wide statistical-genetic analyses, including Mendelian-randomization studies. These refinements did not alter the review question, population-concept-context framework, or requirement for a direct omics-to-irAE analytical link. The operational eligibility definition was set before full-text assessment was completed, and the platform and validation refinements were applied during final data charting before evidence synthesis. Because the purpose was to map the extent, characteristics, and gaps of a heterogeneous evidence base, no meta-analysis or pooled diagnostic-performance estimate was planned.

### 2.2 Eligibility criteria

Eligibility was defined using the population-concept-context framework. The population comprised patients with malignant tumors treated with immune checkpoint inhibitors (ICIs), including agents targeting PD-1, PD-L1, CTLA-4, or other immune checkpoint pathways, as well as combination regimens, or human biospecimens obtained in this setting. The concept was high-throughput omics evidence directly related to an immune-related adverse event (irAE), including susceptibility, occurrence, diagnosis, severity, longitudinal monitoring, clinical course, recovery, response to irAE-directed treatment, or mechanistic characterization. The context encompassed human clinical and translational studies across cancer types, treatment settings, biospecimens, and organ-specific or mixed irAE phenotypes.

Eligible reports were full-text original human studies that generated or analyzed high-throughput omics data and directly linked the resulting molecular features to an irAE outcome. Eligible approaches comprised whole-genome or whole-exome sequencing; genome-wide association, polygenic, or other genome-wide analyses; bulk, single-cell, or spatial transcriptomics; discovery-scale or high-dimensional proteomics; untargeted metabolomics or lipidomics; 16S rRNA gene sequencing or shotgun metagenomics; T-cell or B-cell receptor repertoire sequencing; and integrated multi-omics approaches. Genome-wide statistical-genetic studies, including Mendelian-randomization analyses, were retained as a prespecified conditional category when the outcome represented ICI-related toxicity and the analysis provided an explicit omics-to-irAE link. Patient-level genomic studies and summary-level statistical-genetic analyses were charted separately. Summary-level statistical-genetic findings were interpreted as evidence supporting susceptibility mechanisms or causal hypotheses rather than as validated clinical biomarkers unless replicated directly in an independent patient cohort.

We excluded routine laboratory variables; single cytokines, autoantibodies, metabolites, proteins, or drug levels measured as isolated markers; limited targeted genotyping or HLA testing; small conventional flow-cytometry panels; and other low-dimensional candidate-marker assays. Omics studies were also excluded when they evaluated treatment efficacy without a direct omics-irAE analysis, mentioned toxicity only as background or an undifferentiated safety outcome, involved non-ICI populations, or confined omics experiments to animals or cell models. Reviews, meta-analyses, protocols, guidelines, editorials, commentaries, letters, conference abstracts without a full report, and case reports were excluded. Descriptive case series were excluded when they lacked an analyzable comparator, group-level omics-irAE assessment, or sufficient information to establish a direct association between omics features and irAE outcomes. Reports without sufficient retrievable information for eligibility assessment were also excluded.

### 2.3 Information sources and search strategy

PubMed/MEDLINE, Scopus, Web of Science Core Collection, and Embase constituted the final four-database search. The search covered records from January 1, 2014, and the final database search was completed on July 9, 2026. Each database strategy combined controlled vocabulary and free-text terms for three concepts: immune checkpoint blockade, immune-related adverse events or organ toxicities, and omics technologies or molecular biomarkers. Database-specific syntax and subject headings were adapted to each platform, and no study-design filter was applied. The database searches returned 557 records from PubMed/MEDLINE, 887 from Scopus, 416 from Web of Science Core Collection, and 2,809 from Embase. Complete database-specific strategies, search dates, fields, limits, and record counts are provided in **Supplementary Table 3**.

### 2.4 Study selection and full-text retrieval

All records were imported into Zotero and consolidated into a single screening library. Duplicate detection and identifier-level reconciliation removed 1,151 records from 4,669 search results, leaving 3,518 unique records. Two reviewers independently screened titles and abstracts against the prespecified population, concept, context, and omics eligibility criteria. Potentially eligible reports underwent full-text assessment using the same criteria. Disagreements were resolved through discussion and consensus. Full texts were sought through institutional subscriptions, publisher websites, open-access repositories, Zotero attachments, and other author-accessible sources. Reports that remained unavailable after these retrieval attempts were recorded separately.

The full-text screening library was reconciled using DOI, PMID, standardized title, and bibliographic metadata. Reports without a retrievable full text did not enter the assessed full-text corpus. Each excluded full-text report was assigned one mutually exclusive primary reason. Detailed identifier-level reconciliation, retrieval status, and full-text exclusion decisions are provided in **Supplementary Table 4**.

### 2.5 Data charting

Data were charted in a standardized study-level form. Extracted fields included bibliographic identifiers, publication year, cancer and treatment context, primary irAE phenotype, biospecimen, omics platform, study design, sample size when reported, intended biomarker application, principal molecular features or signatures, data-source type, and validation status. One reviewer completed the initial study-level charting, and a second author independently verified eligibility-critical fields, primary toxicity phenotype, omics-platform assignments, data-source type, and validation status; discrepancies were resolved by consensus after reassessment of the full text. Study-design fields distinguished explicitly reported prospective, retrospective, cross-sectional, case series, and secondary/database analyses, as well as single-center and multicenter recruitment. Biospecimens (blood, tissue or minimally invasive tissue-surface samples, bronchoalveolar lavage fluid, urine, and stool), sampling times (baseline, on-treatment, toxicity onset, and post-treatment), and applications (risk/prediction, diagnosis, longitudinal monitoring, severity/prognostic assessment, mechanistic characterization, and response to irAE-directed treatment or recovery) were coded as non-mutually-exclusive variables. Validation status was classified according to translational maturity. Internal validation comprised resampling, cross-validation, or split-sample procedures conducted within the source cohort. Independent cohort assessment required a separate human cohort and was further classified as either validation of a predefined model/signature or independent replication of individual molecular markers/features. Orthogonal assay confirmation was recorded separately; confirmation in the same cohort, animal experiments, or technical replication without independent patients was not counted as independent cohort validation. Unclear or unreported information was not imputed. For the platform audit, each study was coded using seven binary fields: genomics/statistical genetics, transcriptomics, single-cell/spatial omics, proteomics, metabolomics/lipidomics, microbiome/metagenomics, and immune-repertoire sequencing. Because individual studies could apply multiple technologies, platform assignments were not mutually exclusive and were intended to describe the distribution of omics approaches across the field. Assignments were based on the full text and methods actually applied to human samples or human datasets, rather than the title or a single primary-platform label. Background citations, proposed future analyses, low-dimensional targeted assays, and animal-only omics were not counted. Single-cell RNA sequencing contributed to both the single-cell and transcriptomic fields; repertoire libraries were counted separately. High-dimensional protein assays were counted as proteomics when they were author-described proteomics or met the predefined threshold of profiling at least 50 proteins. To distinguish platform application from genuine molecular integration, we performed an additional assessment of true multi-omics integration. Studies were classified as true multi-omics when they incorporated at least two independent molecular layers within the same study framework. The presence of multiple platform annotations alone was not sufficient for classification as true multi-omics integration. Overlapping analytical modalities were not considered independent molecular layers; for example, single-cell RNA sequencing contributing to both transcriptomics and single-cell/spatial omics was considered a modality overlap rather than a separate multi-omics layer. The completed platform audit, including the true multi-omics assessment, is reported in **Supplementary Table 1**; the methodological-characteristics, genomic evidence-source, and validation audit is reported in **Supplementary Table 2**.

### 2.6 Data synthesis and evidence mapping

The evidence was summarized descriptively by publication year, primary organ-toxicity phenotype, and omics platform. Each study was assigned one primary organ category; studies addressing several irAEs or overall toxicity were classified as mixed/general irAEs. Platform counts were non-mutually exclusive because a single study could contribute to more than one omics domain, whereas true multi-omics integration required at least two independent molecular layers as defined above. An organ-by-platform matrix was constructed from study-level assignments. Selected illustrative findings in **Table 1** were chosen to demonstrate organ coverage, biological coherence, validation status, and translational maturity rather than to rank individual biomarkers or studies. Because of heterogeneity in populations, biospecimens, outcome definitions, analytical workflows, and reported performance measures, no meta-analysis was undertaken. Consistent with the objectives of a scoping review, no formal risk-of-bias or certainty-of-evidence rating was used; instead, evidence maturity was described using study design, validation, longitudinal sampling, and clinical-utility considerations. Study-level counts were reported with explicit denominators. Sample-size summaries were restricted to studies for which the number of ICI-exposed participants in the primary irAE analytic cohort(s) could be unambiguously extracted; summary-level genetic and pharmacovigilance datasets and overlapping cohorts without a defensible participant total were excluded from the sample-size median.

**Table 1:** Selected illustrative organ-by-omics findings and evidence maturity.

Phenotype	Platform	Illustrative signal	Evidence status/caveat
Mixed/general	Plasma proteomics	IL1RL1/FABP3 and multivariable severity model (11)	Independent model/signature validation reported; broader replication needed
Mixed/general	Transcriptomics/single-cell	T-cell, B-cell, myeloid, and interferon-associated states (21, 22, 38, 51)	Predominantly exploratory and cohort-specific
Mixed/general	Microbiome	Baseline and longitudinal community features associated with toxicity (16, 20, 48, 63)	Taxonomic reproducibility and confounding remain concerns
Pneumonitis	Single-cell/transcriptomics	Activated T-cell, monocyte/macrophage, and chemokine programs (12, 56, 59, 70)	Small cohorts; differential-diagnosis validation required
Pneumonitis	Proteomics/miRNA	Extracellular-vesicle proteins and circulating miRNA signatures (15, 29)	Early detection potential; independent validation remains limited overall
Myocarditis	Single-cell/multi-omics	Macrophage expansion, shared clonotypes, and CXCL9/CXCR3 signaling (36, 40, 60, 61)	Mechanistic coherence; event rarity limits prediction models
Colitis	Microbiome/transcriptomics	Barrier dysfunction, tissue-resident cytotoxic T cells, and microbial ecology (13, 42, 50, 93, 96)	Biologically coherent but platform and cohort heterogeneity
Hepatitis	Transcriptomics/repertoire	Exhausted CD8 effector-memory and immune-cell signatures (25, 33)	Only two studies
Renal	Urine proteomics/single-cell	Urinary nephritis signature and circulating immune states (35, 74)	Only two studies; prospective validation required
Dermatologic	Transcriptomics/genomics	Pan-T-cell activation, CXCL10 macrophage signaling, and polygenic susceptibility (68, 75, 94)	Phenotypes and sampling methods heterogeneous
Neurologic	Transcriptomics/repertoire	Clonally expanded cytotoxic CD4 T cells (18)	Single-study evidence
Other rare organs	Mixed omics platforms	Hematologic, myositis, and pancreatic signals (72, 79, 87)	Single-study evidence in each phenotype

## 3 Results

### 3.1 Study selection

The searches identified 4,669 records: PubMed/MEDLINE (n=557), Scopus (n=887), Web of Science Core Collection (n=416), and Embase (n=2,809). After removal of 1,151 duplicates, 3,518 unique records were screened. Of these, 3,085 were excluded at title and abstract screening and 433 reports were sought for retrieval. Thirty-two reports could not be retrieved for full-text assessment and were documented separately as a potential source of retrieval bias, leaving 401 reports assessed for eligibility. A total of 315 full-text reports were excluded: no eligible high-throughput omics platform (n=215), no eligible ICI-irAE population (n=39), no direct irAE outcome (n=26), omics analysis not directly linked to irAE (n=18), non-original research (n=8), preclinical-only omics (n=7), and case report or descriptive case series without an analyzable omics-irAE assessment (n=2). The final review included 86 studies (**Figure 1** and **Table 2**) (11–96).

**Figure 1:**
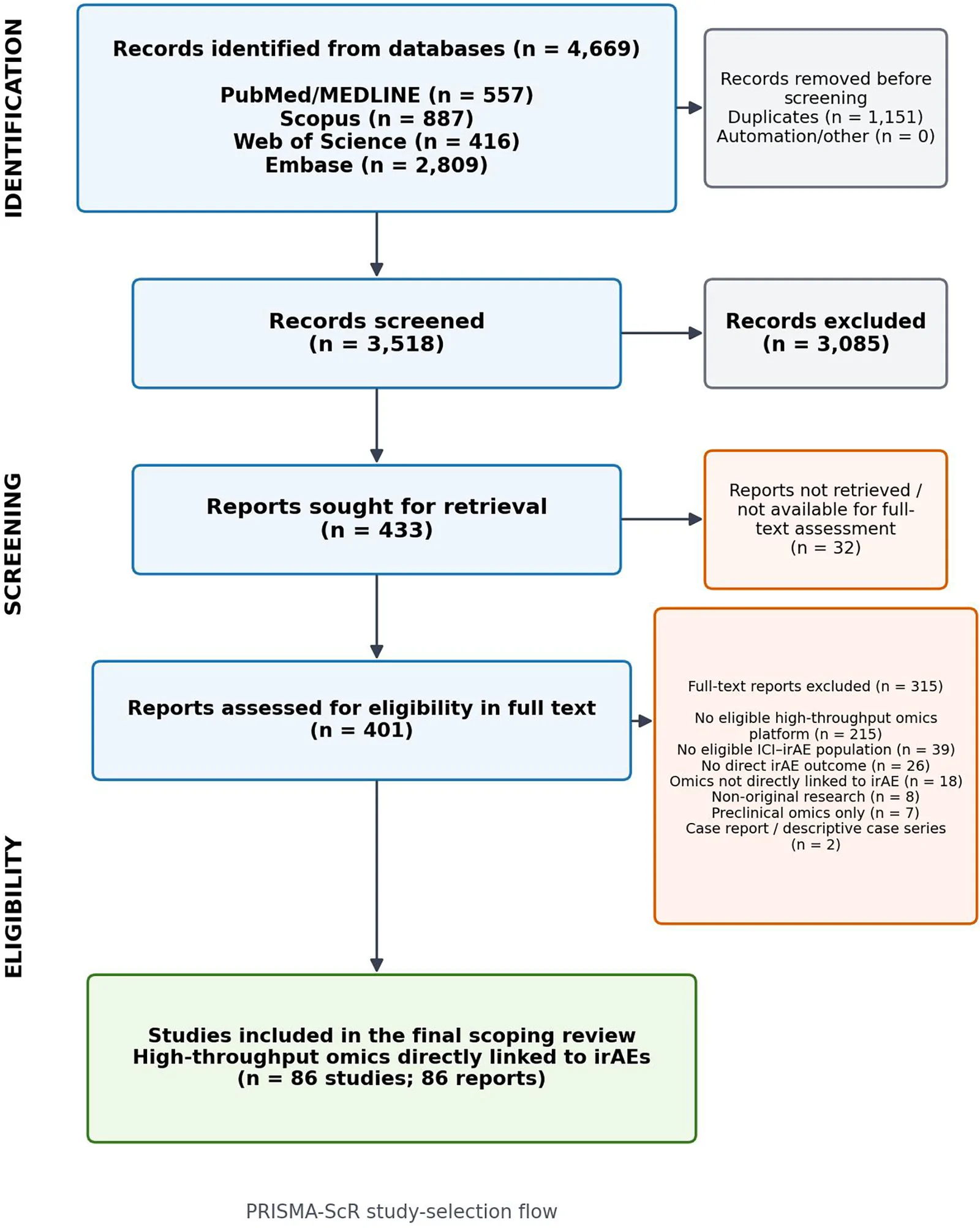
PRISMA-ScR flow diagram for identification, screening, full-text eligibility assessment, and inclusion of high-throughput omics studies directly evaluating immune-related adverse events.

**Table 2:** PRISMA-ScR study-selection summary.

Stage	n
Records identified	4,669
Duplicates removed	1,151
Unique records screened	3,518
Records excluded at title/abstract	3,085
Reports sought for retrieval	433
Reports not retrieved for full-text assessment	32
Reports assessed in full text	401
Full-text reports excluded	315
Studies included	86

### 3.2 Characteristics of included studies

The 86 studies were published from 2017 through 2026 (**Table 3**; **Figure 2**). Publication activity increased sharply in recent years: 66 studies (76.7%) were published from 2023 through July 9, 2026, including 20 in 2024, 20 in 2025, and 12 in 2026. Thirty-nine studies contributed to one classified omics domain and 47 contributed to two or more non-mutually-exclusive platform domains. Because this domain-level overlap included analytical modality overlap, it was not interpreted as equivalent to multi-omics integration. After applying the predefined criterion of at least two independent molecular layers, 35 studies met the criteria for true multi-omics integration. Of the 14 genomics/statistical-genetics studies, 11 used patient-level genomic cohorts or analyses and 3 were summary-level statistical-genetic analyses. The reports explicitly described 24 studies as prospective and 7 as retrospective, with 1 using both prospective and retrospective components; 7 were secondary or database analyses, 4 were case series or cross-sectional studies, and design direction was not stated clearly in 43. Recruitment was explicitly single-center in 24 studies and multicenter in 6; center status was unclear in 56.

**Table 3:** Publication-year distribution.

Publication year	Studies, n	% of 86
2017	1	1.2
2018	1	1.2
2020	3	3.5
2021	7	8.1
2022	8	9.3
2023	14	16.3
2024	20	23.3
2025	20	23.3
2026	12	14.0
2017–2022	20	23.3
2023–2026	66	76.7

**Figure 2:**
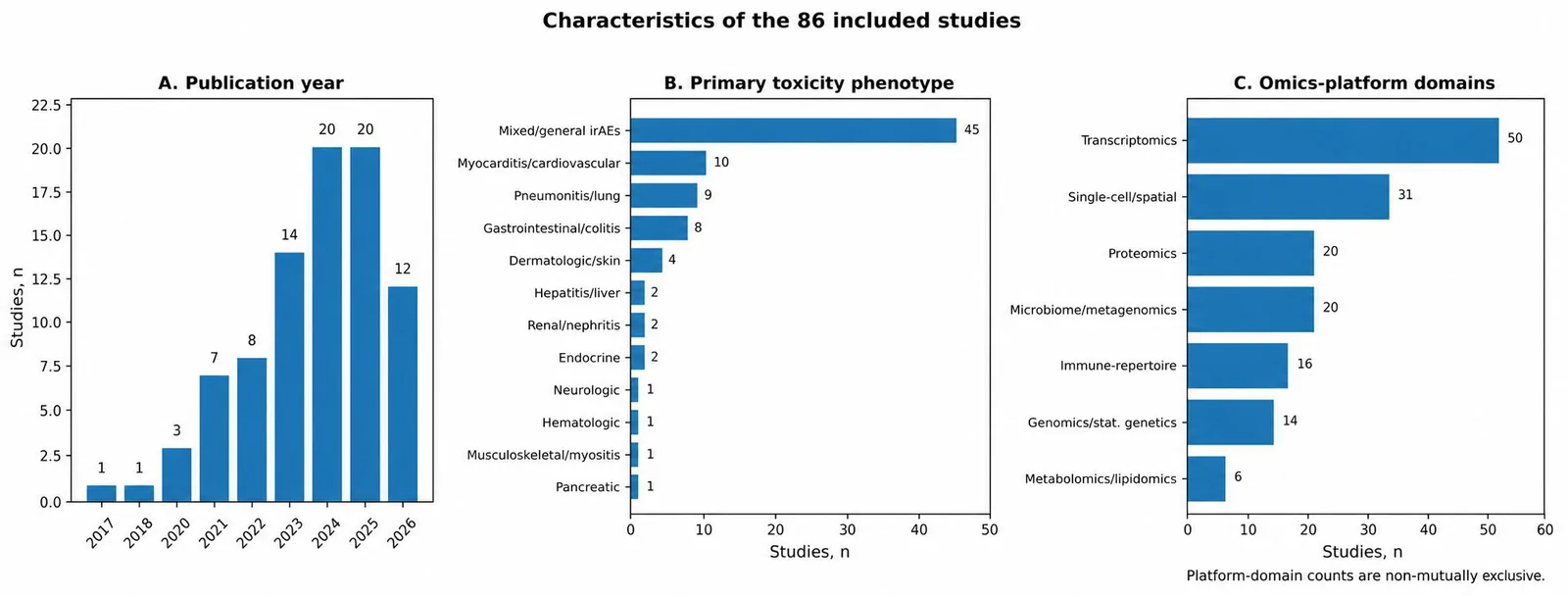
Characteristics of the 86 included studies: **(A)** publication-year distribution; **(B)** primary toxicity phenotype; and **(C)** omics-platform distribution. Platform-domain counts are non-mutually exclusive and should not be interpreted as equivalent to true multi-omics integration.

An unambiguous ICI-exposed analytic-cohort total could be extracted for 66 of the 86 studies. In this subset, the median sample size was 43 participants (range 5–996); 34 studies included 50 or fewer participants, 19 included 51–100, and 13 included more than 100. For the other 20 studies, a participant total was not used because the analysis relied on summary-level data, included overlapping cohorts, or was reported too ambiguously for reliable extraction. Lung cancer was the largest explicitly disease-specific group (n=17), followed by melanoma (n=11), genitourinary cancers (n=3), gastrointestinal/hepatobiliary cancers (n=2), and breast cancer (n=1); the other 52 studies used mixed/pan-cancer populations, other tumor types, or did not restrict the analysis to one clearly reported cancer group. Recruitment was explicitly single-center in 24 studies and multicenter in 6; center status was unclear in 56 (**Table 4**).

**Table 4:** Quantified study-design, biospecimen, timing, application, and validation characteristics.

Domain	Category	Studies, n
Design	Prospective	24
Design	Retrospective	7
Design	Mixed prospective/retrospective	1
Design	Secondary/database	7
Design	Case series/cross-sectional	4
Design	Unclear/not explicitly stated	43
Center	Single-center	24
Center	Multicenter	6
Center	Unclear/not explicitly stated	56
Biospecimen*	Blood/plasma/serum/PBMC	67
Biospecimen*	Stool/fecal	19
Biospecimen*	Tissue/tissue-surface samples	8
Biospecimen*	BALF	5
Biospecimen*	Urine	2
Sampling time*	Baseline/pretreatment	48
Sampling time*	On-treatment/serial	31
Sampling time*	Toxicity onset/diagnosis	22
Sampling time*	Post-treatment/recovery	17
Application*	Risk/prediction/susceptibility	65
Application*	Mechanistic characterization	49
Application*	Longitudinal monitoring/dynamics	30
Application*	Diagnosis/differential-diagnosis assessment	8
Validation*	Internal validation	8
Validation*	Independent cohort assessment	16
Validation*	Independent model/signature validation	2
Validation*	Independent marker/feature replication	14
Validation	Neither internal validation nor independent cohort assessment	65

*Non-mutually-exclusive categories. BALF, bronchoalveolar lavage fluid; PBMC, peripheral blood mononuclear cell.

### 3.3 Biospecimens, sampling time, application, and validation

Biospecimen counts were non-mutually exclusive. Blood-derived material was used in 67 studies, stool in 19, tissue or minimally invasive tissue-surface samples in 8, bronchoalveolar lavage fluid in 5, and urine in 2. Baseline or pretreatment samples were analyzed in 48 studies, on-treatment or serial samples in 31, samples at toxicity onset or diagnosis in 22, and post-treatment or recovery samples in 17. Studies could contribute to more than one time point.

With non-mutually-exclusive application coding, 65 studies evaluated risk, prediction, or susceptibility associations; 49 undertook explicit mechanistic characterization; 30 used longitudinal or dynamic profiling relevant to monitoring; and 8 evaluated diagnostic or differential-diagnosis assessment. These application categories could overlap because some studies generated candidate biomarkers while also investigating biological mechanisms. Internal validation was reported in 8 studies. Independent human cohort assessment was reported in 16 studies, comprising 2 studies with independent validation of a predefined model or signature and 14 with independent replication of one or more molecular markers or features. Altogether, 21 studies reported internal validation and/or independent cohort assessment, whereas 65 reported neither. Orthogonal confirmation in the same cohort was not counted as independent cohort validation. Study-level assignments, genomic evidence type, validation category, and unclear fields are reported in **Supplementary Table 2**. 

### 3.4 Distribution by primary toxicity phenotype

Mixed or general irAEs constituted the largest category (n=45, 52.3%). Among the 41 organ-specific studies, myocarditis or cardiovascular toxicity (n=10), pneumonitis or lung toxicity (n=9), and gastrointestinal toxicity or colitis (n=8) were most frequent. Dermatologic toxicity was represented by four studies; hepatic, renal, and endocrine toxicities by two studies each; and neurologic, hematologic, musculoskeletal/myositis, and pancreatic toxicities by one study each (**Table 5**). Thus, organ coverage was broad, but the evidence remained thin for most less common toxicities.

**Table 5:** Distribution by primary toxicity phenotype.

Primary phenotype	Studies, n	% of 86
Mixed/general irAEs	45	52.3
Myocarditis/cardiovascular	10	11.6
Pneumonitis/lung	9	10.5
Gastrointestinal/colitis	8	9.3
Dermatologic/skin	4	4.7
Hepatitis/liver	2	2.3
Renal/nephritis	2	2.3
Endocrine	2	2.3
Neurologic	1	1.2
Hematologic	1	1.2
Musculoskeletal/myositis	1	1.2
Pancreatic	1	1.2

### 3.5 Distribution by omics platform

Transcriptomics was the most frequently represented domain (n=50), followed by single-cell or spatial omics (n=31), proteomics (n=20), microbiome or metagenomic profiling (n=20), immune-repertoire sequencing (n=16), genomics or statistical genetics (n=14), and metabolomics or lipidomics (n=6). Counts exceed 86 because platform-domain assignments were non-mutually exclusive; 47 studies contributed to two or more domains, which should not be interpreted as equivalent to true multi-omics integration. Single-cell RNA sequencing contributed to both the transcriptomic and single-cell/spatial domains by design, whereas immune-repertoire sequencing was coded as a separate molecular layer. Microbiome studies were concentrated in mixed irAEs and gastrointestinal toxicity (**Table 6** and **Figure 3**).

**Table 6:** Omics-platform distribution.

Omics platform	Studies, n	% of 86
Transcriptomics	50	58.1
Single-cell/spatial omics	31	36.0
Proteomics	20	23.3
Microbiome/metagenomics	20	23.3
Immune-repertoire sequencing	16	18.6
Genomics/statistical genetics	14	16.3
Metabolomics/lipidomics	6	7.0

**Figure 3:**
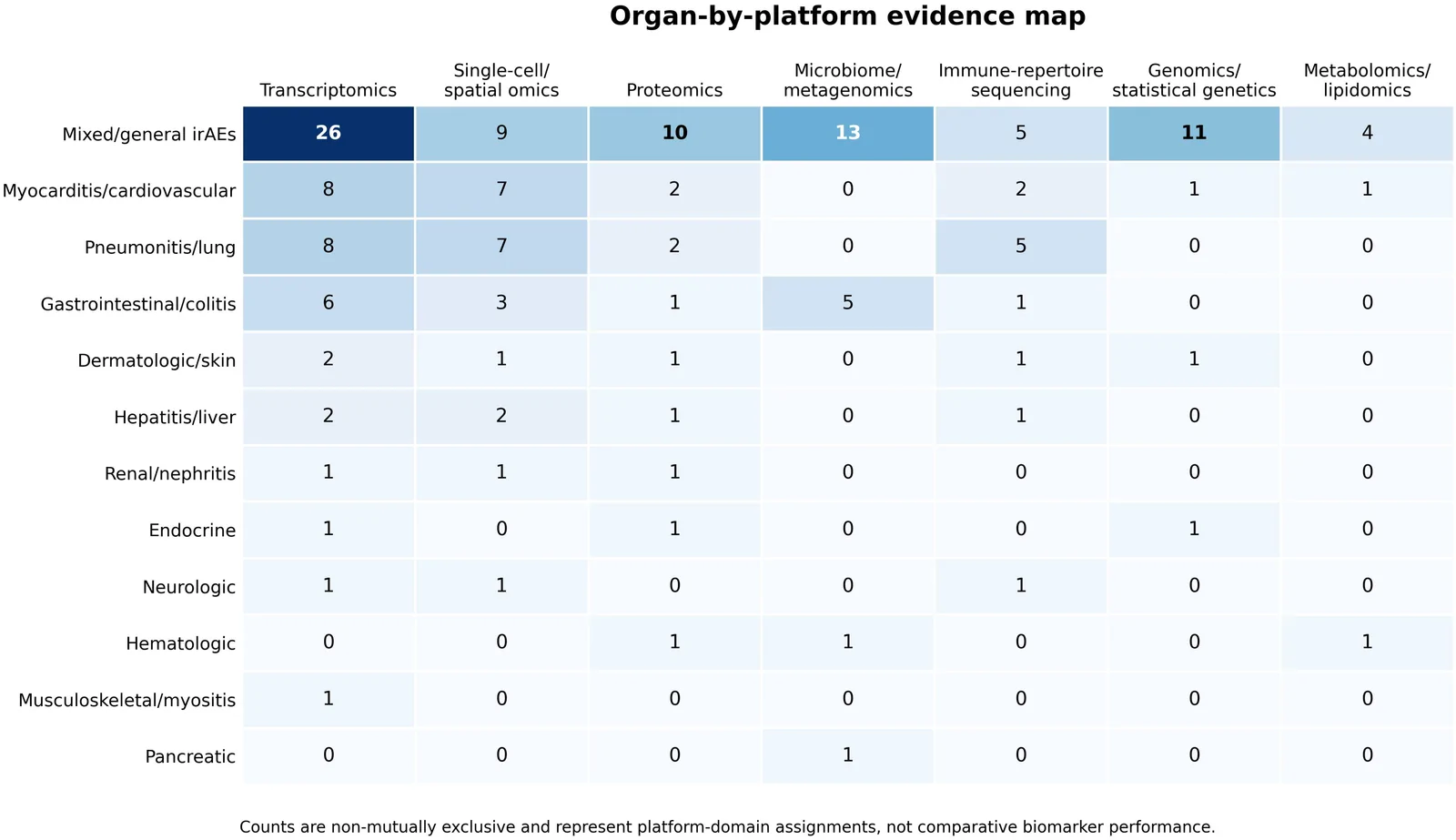
Organ-by-platform evidence map. Each cell represents the number of included studies assigned to a specific primary irAE phenotype and omics domain. Counts are non-mutually exclusive because individual studies may contribute to multiple domains. The map illustrates evidence distribution rather than comparative biomarker performance or clinical utility; platform-domain overlap should not be interpreted as equivalent to true multi-omics integration.

### 3.6 Organ-specific molecular patterns

#### 3.6.1 Mixed and general irAEs

The 45 mixed/general studies used every classified platform. Transcriptomic and single-cell studies identified baseline or treatment-emergent immune states involving T-cell activation, regulatory T-cell reprogramming, B-cell dynamics, myeloid programs, and interferon-associated signaling (21, 22, 24, 26, 32, 38, 39, 45, 46, 49, 51, 66, 78, 83, 95). Proteomic studies described circulating immune and tissue-injury signatures, including plasma models for irAE occurrence and severity, while genome-wide and statistical-genetic analyses evaluated inherited susceptibility and causal protein or microbial candidates (11, 23, 27, 31, 52, 55, 57, 64, 67, 69, 84, 88). Microbiome studies linked baseline community structure and longitudinal microbial changes with subsequent toxicity, although taxa and effect directions varied across cohorts (16, 20, 48, 53, 63, 71, 73, 76, 81, 82, 85, 91, 92). Metabolomic or lipidomic profiling contributed additional circulating and microbiome-related signals in mixed/general cohorts (19, 23, 39, 76).

#### 3.6.2 Pneumonitis and lung toxicity

The nine pneumonitis studies were dominated by single-cell and transcriptomic approaches. Bronchoalveolar and tissue analyses repeatedly implicated activated or clonally expanded T-cell states, inflammatory monocytes and macrophages, antigen-presentation programs, interferon signaling, and chemokine networks (12, 14, 17, 56, 59, 70, 89). Circulating microRNA and extracellular-vesicle proteomic studies explored less invasive prediction and early detection (15, 29). The pattern is consistent with compartment-specific immune injury, although most cohorts were small and validation against infection, radiation pneumonitis, and tumor progression remains necessary.

#### 3.6.3 Myocarditis and cardiovascular toxicity

Ten studies addressed myocarditis or cardiovascular toxicity. Single-cell, tissue transcriptomic, and immune-repertoire analyses identified pathogenic macrophage expansion, activated cytotoxic lymphocytes, shared heart-blood-tumor immune clones, CXCL9/CXCR3 signaling, and inflammatory fibroblast or endothelial states (28, 34, 36, 40, 43, 60, 61, 77). One multi-omic study also included proteomic and lipidomic profiling (43), while additional proteomic and genomic studies examined mitochondrial metabolism and clonal hematopoiesis as complementary mechanistic or susceptibility signals (44, 90). Similar signals across tissues strengthen the biological interpretation, but model development remains constrained by the rarity of myocarditis, variable diagnostic adjudication, and small case numbers.

#### 3.6.4 Gastrointestinal toxicity and colitis

The eight gastrointestinal/colitis studies combined mucosal or single-cell transcriptomics with microbiome profiling. Recurrent themes included epithelial-barrier dysfunction, tissue-resident cytotoxic T-cell activation, interferon signaling, host-microbial interactions, and microbial community features associated with susceptibility or treatment response (13, 42, 50, 54, 62, 86, 93, 96). Fecal microbiota transplantation studies also provided proof-of-concept that the microbiome may be therapeutically modifiable (42). Nevertheless, cohort-specific microbial signatures, antibiotic and dietary confounding, and variation in stool collection and sequencing pipelines impede reproducibility.

#### 3.6.5 Less-represented organ toxicities

Hepatic studies identified circulating exhausted CD8 effector-memory states and combined transcriptomic, immune-repertoire, or protein features (25, 33). Renal studies used urine proteomics and peripheral-blood single-cell transcriptomics (35, 74). Dermatologic studies evaluated tissue or tape-strip transcriptomics, single-cell macrophage signaling, proteomic profiles, and polygenic susceptibility (47, 68, 75, 94). Endocrine evidence included genetic susceptibility and omics characterization of ICI-associated diabetes (41, 80). The single hematologic study combined gut metagenomics with plasma metabolomics and proteomics (72). Only one study each addressed neurologic toxicity, hematologic toxicity, myositis, and pancreatic toxicity (18, 72, 79, 87), highlighting major gaps in organ coverage.

### 3.7 Evidence maturity and limitations

Validation evidence remained limited. Twenty-one of the 86 studies reported internal validation and/or independent cohort assessment; 16 included an independent human cohort, but only 2 evaluated a predefined model or signature in that setting, whereas 14 replicated individual molecular markers or features. Among the 66 studies with extractable participant totals, 34 (51.5%) enrolled 50 or fewer participants. Only 6 studies were explicitly multicenter, while center status was unreported or unclear in 56. Baseline sampling was more frequent (n=48) than toxicity-onset sampling (n=22) or post-treatment/recovery sampling (n=17). Taken together, the evidence remains largely exploratory: cohorts were often small, independent model-level validation was uncommon, and design direction and center structure were frequently reported incompletely. Heterogeneous irAE definitions, incomplete adjustment for cancer type and concomitant therapy, and limited clinical-utility testing further constrained interpretation. No omics signature had sufficient replicated evidence and demonstrated clinical utility to support routine implementation.

## 4 Discussion

### 4.1 Principal findings

This scoping review identified 86 original human studies in which high-throughput omics features were directly related to irAEs. The literature has expanded rapidly since 2023 and is dominated by transcriptomic and single-cell approaches. Although 47 studies contributed to two or more omics domains in the platform audit, this number reflects platform application rather than definitive molecular-layer integration. After accounting for overlapping analytical modalities and requiring independent molecular layers, 35 studies met the predefined criteria for true multi-omics integration. The methodological audit showed that validation evidence remained limited: 21 studies reported internal validation and/or independent cohort assessment, 16 included assessment in an independent human cohort, and only 2 evaluated a predefined model or signature in that setting. The median ICI-exposed analytic-cohort size was 43 (range 5-996) among 66 studies with an extractable participant total. Metabolomics/lipidomics remained relatively underrepresented, with six identified studies. Nevertheless, more than half of the evidence addressed mixed or general irAEs, and organ-specific evidence remained concentrated in myocarditis, pneumonitis, and colitis. Most reported signatures remain exploratory and have not progressed through rigorous independent model-level validation or clinical-utility evaluation.

### 4.2 Comparison with previous reviews

Previous reviews summarized broad candidate biomarkers and clinical risk factors, including routine laboratory measures, circulating cytokines, autoantibodies, HLA variants, immune-cell counts, and the gut microbiome (1–7). That breadth is clinically informative but obscures the size and maturity of the high-throughput omics literature. By requiring both an eligible omics platform and a direct analytical link to an irAE outcome, the present review delineates a smaller, more methodologically coherent evidence base. The map separates primary organ phenotypes, distinguishes overlapping platform-domain assignments from true multi-omics integration, and separates patient-level genomic evidence from summary-level statistical genetics. These features make the current evidence map complementary to previous broad biomarker reviews and highlight how strongly discovery studies continue to outnumber studies with mature independent validation.

### 4.3 Interpretation of cross-organ molecular patterns

Across different organ toxicities, several studies reported recurrent molecular patterns involving activated or clonally expanded T-cell states, myeloid activation, interferon and chemokine signaling, and loss of tissue-specific immune homeostasis. However, these apparent similarities should be interpreted cautiously because they may partly reflect repeated use of similar technologies, biospecimens, analytical pipelines, or potentially overlapping patient cohorts. The relevant cellular compartment also differed by phenotype: pneumonitis studies emphasized bronchoalveolar T cells and inflammatory monocytes; myocarditis studies highlighted cardiac macrophages, cytotoxic lymphocytes, stromal activation, and shared clonotypes; and colitis studies linked mucosal immune injury to epithelial-barrier dysfunction and microbial ecology. Independent replication across different cohorts, platforms, and clinical settings is required to determine which recurring signals represent genuine biological convergence.

A shared background of checkpoint-release-induced immune activation remains biologically plausible, but tissue-specific cellular niches are likely to modify how toxicity manifests. Clonally expanded cytotoxic lymphocytes and interferon-associated programs recur across several organs, whereas macrophage-stromal interactions are especially prominent in myocarditis, epithelial-microbial interactions in colitis, and bronchoalveolar myeloid-T-cell crosstalk in pneumonitis. For biomarker development, these observations support testing combinations of shared and organ-specific features rather than assuming a single universal irAE signature.

Mixed-irAE studies may be useful for identifying common susceptibility states before treatment, but pooling biologically heterogeneous toxicities can introduce noise, dilute organ-specific signals, and produce models that are difficult to interpret clinically. Future analyses should therefore distinguish pan-irAE risk prediction from organ-specific diagnosis and severity assessment and should report both aggregate and phenotype-specific performance whenever sample size permits.

### 4.4 Translational readiness

Clinical use will require more than an association: a candidate test must be prespecified, analytically reproducible, and actionable. Candidate signatures should demonstrate analytical validity, incremental value beyond clinical variables, temporal precedence over toxicity when intended for prediction, and rigorous independent validation. The present audit further shows why validation terminology matters: replication of an individual molecular feature in an independent cohort is not equivalent to evaluation of a predefined multivariable model or threshold. Most studies in this review satisfied only discovery or early clinical-association stages, and reported performance estimates were often derived from high-dimensional datasets with few events, making overfitting a major concern. Orthogonal confirmation of selected features is valuable but does not substitute for validation of a complete model in an independent population. Summary-level Mendelian-randomization or other statistical-genetic findings may inform susceptibility mechanisms or causal hypotheses, but they should not be interpreted as validated patient-level biomarkers without direct clinical replication. Additional barriers to translation include assay standardization, harmonized sample processing, multicenter prospective validation, regulatory qualification, cost, and integration into clinical decision workflows.

Near-term applications will probably depend on when and where samples are collected. Baseline blood, germline, and microbiome features may support susceptibility assessment; serial blood or stool profiles may enable on-treatment surveillance; and tissue, bronchoalveolar, urine, or organ-specific molecular profiles may assist diagnosis when toxicity occurs. A clinically useful strategy will probably combine parsimonious omics-derived features with symptoms, routine laboratory variables, imaging, and adjudicated organ phenotypes rather than deploy an omics signature in isolation.

### 4.5 Methodological priorities for future research

Prospective multicenter cohorts should use harmonized irAE definitions, severity grading, diagnostic adjudication, and biospecimen protocols. Sampling should include pretreatment, early on-treatment, toxicity-onset, treatment, recovery, and rechallenge time points. Controls should distinguish ICI-treated patients without toxicity from patients with infection, cancer progression, treatment-related nonimmune injury, and relevant autoimmune diseases. Studies should prespecify analytical pipelines, handle batch effects and missing data transparently, report calibration and decision-curve analysis in addition to discrimination, and evaluate whether the model adds value beyond readily available clinical predictors.

Rare toxicities will require collaborative registries with nested biospecimen studies. Resources such as the Chinese Tumor Immune-related Organ Injury Network (C-TION) and the Intelligent Data Platform for Tumor Immunorelated Medical Information Mart of China (TIMIC) could support harmonized phenotyping and multicenter analyses of these uncommon events. Endocrine, renal, neurologic, hematologic, pancreatic, musculoskeletal, and severe dermatologic irAEs were particularly underrepresented. Common data models and federated analyses may provide adequate event numbers without transferring sensitive molecular data. Multi-omics integration should be driven by a clear biological or clinical question and accompanied by dimensionality reduction, internal resampling, independent validation, and accessible reporting of features and code. Complementary large-scale pharmacovigilance can also help prioritize rare but high-consequence toxicities for molecular investigation: a recent analysis combining FAERS and VigiBase with additional clinical datasets identified ICI-associated adverse events with disproportionately high reported fatality, including interstitial lung disease, myositis, and hepatic failure (97). Such signals are useful for prioritization but do not replace mechanistic or predictive biomarker validation because spontaneous-reporting data remain susceptible to reporting bias and incomplete clinical annotation.

### 4.6 Strengths and limitations of this review

The review used a coordinated four-database search, an operational definition of high-throughput omics, duplicate and identifier-level reconciliation, full-text reassessment with mutually exclusive exclusion reasons, second-author verification of study-level assignments, and a cross-organ evidence map. Platform and organ counts were derived from the final 86-study corpus using a non-mutually-exclusive, seven-domain platform audit.

This review also has limitations. The search terminology included broad molecular-biomarker terms to maximize sensitivity, generating a large number of non-omics full-text exclusions. Thirty-two reports could not be retrieved for full-text assessment despite multiple retrieval attempts; this may have introduced retrieval bias if unavailable reports differed systematically in organ phenotype, omics platform, or study design, and the available bibliographic information was insufficient to determine whether their inclusion would materially alter the organ-by-platform distribution. Platform categories overlap, and assignment of one primary organ phenotype simplifies studies that assessed several toxicities. Mixed/general irAE analyses may identify broad susceptibility states but can obscure tissue-specific molecular drivers. Apparent cross-organ convergence may also partly reflect repeated use of similar technologies, biospecimens, analytical pipelines, or overlapping cohorts. Because cohort provenance was inconsistently reported, we could not reliably quantify the extent of patient-cohort overlap across publications. Patient-level molecular studies and summary-level statistical-genetic analyses were mapped within a common platform framework even though they represent distinct evidence types; this distinction was retained in study-level charting and interpretation. The review did not perform a formal risk-of-bias assessment or meta-analysis, and evidence maturity was summarized descriptively. Reporting of cohort size, biospecimen timing, irAE adjudication, and validation was inconsistent across studies. Finally, the rapidly evolving 2025–2026 literature means that the map will require periodic updating.

## 5 Conclusion

High-throughput omics research directly evaluating immune-related adverse events is expanding rapidly but remains predominantly exploratory. Among 86 included studies, transcriptomics and single-cell approaches were most common; 47 studies contributed to multiple platform domains, whereas 35 met the predefined criteria for true multi-omics integration involving independent molecular layers. Metabolomics/lipidomics was represented in six studies, mixed/general irAEs accounted for more than half of the evidence, and myocarditis, pneumonitis, and colitis dominated organ-specific research. Although 16 studies included independent human cohort assessment, only 2 evaluated a predefined model or signature in an independent cohort, and clinical-utility testing was rare. Before implementation, candidate markers require testing in prospective multicenter cohorts with standardized phenotyping, longitudinal sampling, transparent model development, and organ-specific independent validation. Future integration of multiple molecular layers with advanced computational approaches, including artificial intelligence and machine learning, may improve multimodal feature selection and individualized risk stratification, but transparent development, independent validation, and clinically meaningful endpoints remain essential.

## Data Availability

The data supporting the conclusions of this article are included in the article and its **Supplementary Material**. Additional review materials are available through the Open Science Framework at https://osf.io/g79cv.
